# Backbone and nearly complete side-chain chemical shift assignments of the human death-associated protein 1 (DAP1)

**DOI:** 10.1007/s12104-020-09988-x

**Published:** 2020-12-02

**Authors:** Christoph Wiedemann, Johanna Voigt, Jan Jirschitzka, Sabine Häfner, Oliver Ohlenschläger, Frank Bordusa

**Affiliations:** 1 Institute of Biochemistry and Biotechnology, Charles Tanford Protein Centre, Martin Luther University Halle-Wittenberg, Kurt-Mothes-Str. 3a, 06120 Halle, Germany; 2 Department of Chemistry, Institute of Biochemistry, University of Cologne, Zülpicher Str. 47, 50674 Cologne, Germany; 3 Leibniz Institute on Aging - Fritz Lipmann Institute, Beutenbergstr. 11, 07745 Jena, Germany

**Keywords:** Death-associated protein 1, Human, Intrinsically disordered protein, IDP, Cell growth, Cell migration, Autophagy, Apoptosis, resonance assignment, Chemical shifts

## Abstract

Death-associated protein 1 (DAP1) is a proline-rich cytoplasmatic protein highly conserved in most eukaryotes. It has been reported to be involved in controlling cell growth and migration, autophagy and apoptosis. The presence of human DAP1 is associated to a favourable prognosis in different types of cancer. Here we describe the almost complete ${{^{1}}\text {H}}$, ${{^{13}}\text {C}}$, and ${{^{15}}\text {N}}$ chemical shift assignments of the human DAP1. The limited spectral dispersion, mainly in the ${{^{1}}\text {H}{^{\text{N}}}}$ region, and the lack of defined secondary structure elements, predicted based on chemical shifts, identifies human DAP1 as an intrinsically disordered protein (IDP). This work lays the foundation for further structural investigations, dynamic studies, mapping of potential interaction partners or drug screening and development.

## Biological context

**Fig. 1:**
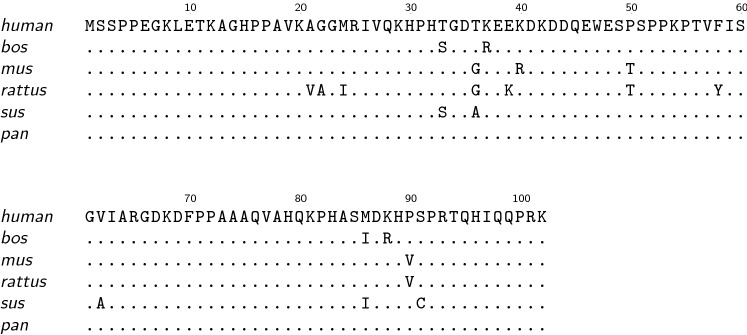
Sequence alignment of Death-associated proteins 1 from selected species. The human DAP1 sequence is used as consensus. Identical residues in other sequences are blanked out, mismatches are shown

The human Death-associated protein 1 (DAP1) is a member of the DAP family (DAP1-5)—originally identified as a diverse group of proteins that constitute biochemical pathways leading to apoptosis (Levy-Strumpf and Kimchi 1998). DAP1 is highly conserved in most eukaryotes and ubiquitously expressed in many cells and tissues. It was originally discovered in HeLa-cells, which were under the constant influence of apoptosis-inducing IFN-γ (Deiss et al. 1995). The high sequence homology especially within higher eukaryotes (Fig. 1) raises the question whether DAP1 is a young protein in evolutionary terms or whether the strong sequence conservation is a prerequisite for a fundamental function of DAP1 identical in all higher organisms.

The human *DAP1* gene encodes for a cytoplasmatic protein (UniProtKB - P51397) of 102 amino acid with a proline content of nearly 15%. Human cell line experiments identified DAP1 as a phosphoprotein (S3 and S51 are phosphorylated) under nutrient-rich conditions. However, stress conditions trigger rapid dephosphorylation of DAP1 (Koren et al. 2010b). The downstream effectors of DAP1 are still subject of intensive research (Yahiro et al. 2014; Nie et al. 2020), but a proposed candidate for upstream regulation is mTOR (Koren et al. 2010a, b). The important role of mTOR in cell proliferation and metabolism is well known and DAP1 is constantly phosphorylated by mTOR due to its SerineThreonine kinase activity under normal conditions. The interplay between de- and phosphorylation is hypothesized to be a key factor for the activity of DAP1. Koren et al. (2010a, b) identified dephosphorylated DAP1 as a suppressor of autophagy and as a novel substrate of mTOR. DAP1 is rapidly activated by dephosphorylation upon inactivation of mTOR, so that the suppressive influence of dephosphorylated DAP1 acts as an antagonist to the autophagic flux (Koren et al. 2010a, b). Lacking any functional motif the role and precise mechanism of DAP1 in autophagy is still poorly understood.

Displaying a general regulatory effect on cellular growth DAP1 seems to have an inhibitory effect on cell migration, autophagy and apoptosis (Koren et al. 2010b; Wazir et al. 2012; Xia et al. 2017; Yahiro et al. 2014). An imbalance in autophagy leads to the formation of different types of tumours (Udristioiu and Nica-Badea 2019). In this context a connection between DAP1, autophagocytosis and human carcinogenesis has been discovered (Wazir et al. 2015). The presence of DAP1 is associated to a favorable prognosis in breast, ovarian, colorectal cancer and others (Wazir et al. 2015; Nie et al. 2020; Jia et al. 2014), even though the positive influence is still under discussion in the literature (Santos et al. 2015).

Its role in cellular growth, programmed cell death and autophagy renders DAP1 an interesting target for future structural and/or interaction studies with regard to potential drug screening and development. But so far, there are no biophysical or structural studies available in literature. Here we report the almost complete ${^{1}\text {H}}$, ${^{13}\text {C}}$, and ${^{15}\text {N}}$ backbone and side chain resonance assignments of the human DAP1.

## Methods and experiments

### Protein expression and purification

The full-length human *DAP1* gene, codon optimized for expression in *E. coli*, was ordered from Thermo Fischer Scientific (Germany) and subcloned with NdeI and XhoI restriction enzymes into a pET28a expression vector, providing an N-terminal ${\text {His}_{6}}$ tag. Subcloning was confirmed by DNA sequencing (Eurofins Genomics, Germany). The construct pET28a-${\text {His}_{6}}$-hDAP1 was transformed in *E. coli* BL21(DE3) cells and plated onto kanamycin plates. A single colony from the plate was picked and grown in LB-Medium (supplemented with 50 μg/ml kanamycin) at 37 °C until OD$_{\text{600\,nm}}$ reached 0.7. Cells were pelleted at 5250xg for 20 min using a Beckman Coulter SX4750A swinging bucket rotor, subsequently washed with 20 ml PBS (phosphate buffered saline) and pelleted again. After resuspension in 250 ml M9 mineral salts medium supplemented with 1 g/l ${{^{15}}\text {NH}_{4}\text {Cl}}$ and 4 g/l ${{^{13}}\text {C}_{6}}$-labeled glucose, gene expression was induced by adding 1 mM IPTG (isopropyl-1-thio-D-galactopyranoside) at 37 °C to the bacteria culture. After 3 h the *E. coli* cells were harvested and stored at − 20 °C. For human DAP1 protein purification the frozen cells were resuspended in buffer (50 mM ${\text {Na}_{2}\text {HPO}_{4}}$, pH 8, 300 mM NaCl, 10 mM imidazole), lysed with sonification and then centrifuged at 10,000×*g* for 15 min. The clear supernatant was applied to Ni-NTA affinity chromatography. The resin was washed with at least 10 column volumes each of 10 mM and 20 mM imidazole containing lysis buffer. Purified human DAP1 was eluted with 0.25 M imidazole and subsequently further purified on a 16/60 HiLoad S75 size exclusion chromatography column (GE Healthcare) with 10 mM ${\text {Na}_{2}\text {HPO}_{4}}$, pH 6.5, 150 mM NaCl. The fractions containing human DAP1 were pooled together and concentrated. The purity of the obtained protein was additionally confirmed by SDS-PAGE. The final concentration of the human DAP1 NMR sample was about 0.8 mM.

We want to mention that the used construct has a thrombin cleavage site between the N-terminal ${\text {His}_{6}}$ tag and the native human DAP1 sequence. Although no further thrombin cleavage site is predicted for the native human DAP1, the addition of thrombin results not only in the cleavage of the N-terminal ${\text {His}_{6}}$ tag but also in a construct shortened by 9 amino acids at the C-terminus (cleavage after R93). Therefore the removal of the purification tag was waived and the amino acid numbering is as follows: − 19 to 0 indicates the purification tag and the native human DAP1 sequence starts with methionine number 1.

### NMR spectroscopy

All NMR experiments for ${^{1}\text {H}}$, ${^{15}\text {N}}$ and ${^{13}\text {C}}$ chemical shift assignments were acquired at 10 °C in 10 mM ${\text {Na}_{2}\text {HPO}_{4}}$, pH 6.5, 150 mM NaCl (90 % H2O/10 % D2O) on a Bruker Avance III NMR system equipped with a 5 mm TXI triple resonance probe and a magnetic field strength of 16.4 T, corresponding to a ${^{1}\text {H}}$ resonance frequency of 700.5 MHz.

Backbone chemical shifts were assigned from a series of spectra including 2D [$^{1}\text {H}$, $^{15}\text {N}$]-HSQC and the Band-selective Excitation Short-Transient (BEST) version of the following standard 3D experiments: HNCO, HN(CA)CO, HNCA, HN(CO)CA and HNCACB (Schanda et al. 2006; Lescop et al. 2007). The bandwidth of the shaped ${^{1}\text {H}}$ pulses was 5 ppm and the offset was set to 8.3 ppm. The inter-scan delay was set to 200 ms. Side chain assignments were obtained by analysis of spectra including 2D constant-time [$^{1}\text {H}$, $^{13}\text {C}$]-HSQC, [$^{1}\text {H}$, $^{15}\text {N}$]-TOCSY-HSQC, HCCH-COSY, HCCH-TOCSY, CC(CO)NH and H(CCCO)NH. The sequential assignment, mainly of the proline residues, was accompanied by ${^{13}\text {C}}$-detected 2D CON and CACO and a series of additional 3D experiments using HANH, HA(CO)NH, (H)N(COCA)NNH, H(NCOCA)NNH, (HCA)CON(CA)H, HACACO, HACAN, HACA(CO)N. Sequence-specific side chain assignments of the aromatic residues were obtained from 2D aromatic [$^{1}\text {H}$, $^{13}\text {N}$]-HSQC, (HB)CB(CGCD)HD and (HB)CB(CGCDCE)HE experiments. All applied experiments are implemented in the Bruker Topspin pulse catalogue and applied without any further modification. Data acquisition and processing was performed with Topspin 3.6.2 (Bruker Biospin GmbH, Rheinstetten). The 3D experiments were recorded with 25% non-uniform sampling (NUS) and Multi-Dimensional Decomposition (MDD) was used for data reconstruction (Orekhov and Jaravine 2011). The spectra were analyzed using CCPNmr Analysis 2.5 (Vranken et al. 2005) within the NMRbox virtual environment (Maciejewski et al. 2017).

${^{1}\text {H}}$ resonances were calibrated with respect to the signal of 2,2-dimethylsilapentane-5-sulfonic acid (DSS). ${^{13}\text {C}}$ and ${^{15}\text {N}}$ chemical shifts were referenced indirectly to the ${^{1}\text {H}}$ standard (Wishart et al. 1995).

**Fig. 2:**
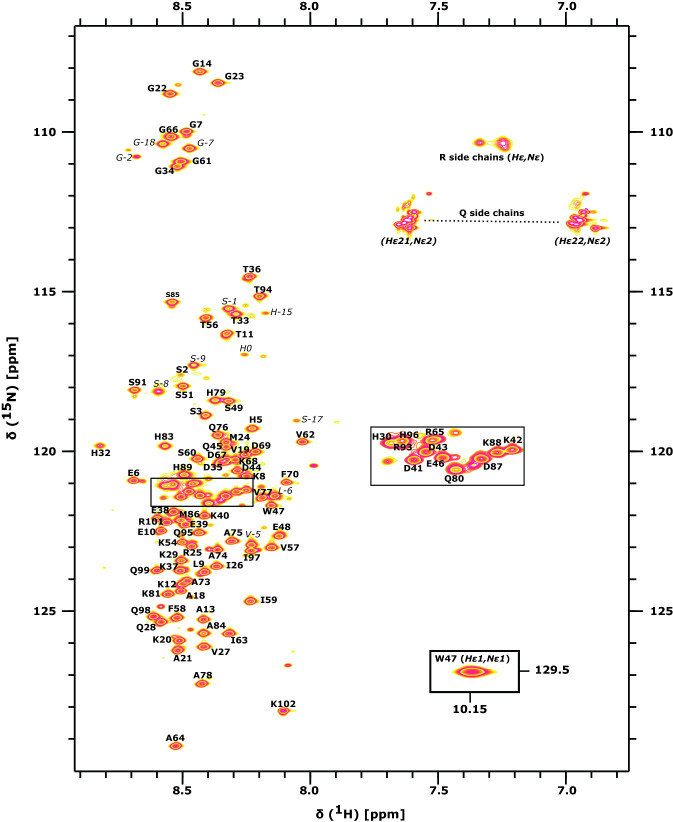
[$^{1}\text {H}$, $^{15}\text {N}$]-HSQC spectrum of ${^{13}}\text {C}$,${^{15}}\text {N}$-labeled human DAP1 at pH 6.5, 10 °C. Assignments for backbone amides are annotated in bold face. Non-degenerate protons of the side chain amino groups are connected by a dashed line. Assignable resonances originating from the N-terminal purification tag are marked in italic

**Fig. 3:**
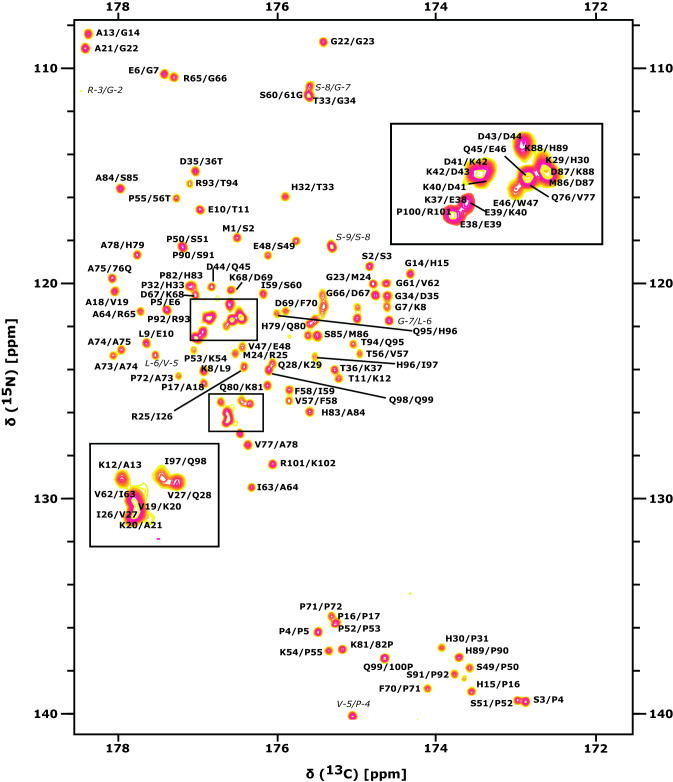
[${^{13}}\text {CO}$, ${^{15}}\text {N}$]-spectrum of ${^{13}}\text {C}$,${^{15}}\text {N}$-labeled human DAP1 at pH 6.5, 10 °C. Assignments for backbone ${^{13}\text {CO}}$, ${^{15}\text {N}}$ correlations of neighboring residues are annotated in bold face. Assignable resonances originating from the N-terminal purification tag are marked in italic

### Structure prediction

For the sequence-based prediction of disordered protein regions the IUPred2A server was used (Dosztányi 2018; Mészáros et al. 2018). Since no essential structural elements were predicted for human DAP1 we could assume that it is an intrinsically disordered protein. Therefore, the random coil chemical shifts of human DAP1 were calculated using POTENCI (Nielsen and Mulder 2018). The potential secondary structure elements of human DAP1 were analyzed by applying the NMR chemical shifts with the web server CSI 3.0 (Hafsa et al. 2015). The secondary structure propensity was examined with the approach provided by Ja et al. (2006).

## Extent of assignments and data deposition

Sequence specific resonance assignments of human DAP1 could be carried out for nearly all ${^{1}\text {H}}$, ${^{13}\text {C}}$ and ${^{15}\text {N}}$ spins using the suite of 2D and 3D NMR experiments mentioned in Methods and Experiments 2.2. The extent of assignment is summarized in Table 1. The $^{1}\text {H}$,$^{15}\text {N}$ assignments obtained are indicated in the [${^{1}\text {H}}$, ${^{15}\text {N}}$]-HSQC spectrum of human DAP1 (Fig. 2). The backbone ${^{13}\text {CO}}$,${^{15}\text {N}}$-correlations of neighboring residues in the 2D CON experiment is given in Fig. 3.

**Table 1:** Extent of backbone and side chain assignment of human DAP1

Nucleus	Assigned (%)	Total number
${{^{1}}\text {H}^{\text{N}}}$	99	86 out of 87
${{^{15}}\text {N}^{\text{H}}}$	99	101 out of 102
${{^{13}}\text {CO}}$	100	102 out of 102
${{^{1}}\text {H}^{\alpha }}$	100	109 out of 109
${{^{1}}\text {H}^{\beta }}$	98	163 out of 167
${{^{1}}\text {H}^{\gamma }}$	98	120 out of 122
${{^{1}}\text {H}^{\delta }}$	88	69 out of 78
${{^{1}}\text {H}^{\epsilon }}$	50	28 out of 56
${{^{13}}\text {C}^{\alpha }}$	100	102 out of 102
${{^{13}}\text {C}^{\beta }}$	100	95 out of 95
${{^{13}}\text {C}^{\gamma }}$	81	71 out of 88
${{^{13}}\text {C}^{\delta }}$	66	40 out of 61
${{^{13}}\text {C}^{\epsilon }}$	65	17 out of 26

The [${^{1}\text {H}}$, ${^{15}\text {N}}$]-HSQC spectrum shows a limited signal dispersion in the ${^{1}\text {H}}$ dimension typically observed for highly flexible or intrinsically disordered proteins. The obtained human DAP1 chemical shifts assignment was validated against the sequence based predicted random coil chemical shifts for intrinsically disordered proteins from the POTENCI web server.

**Fig. 4:**
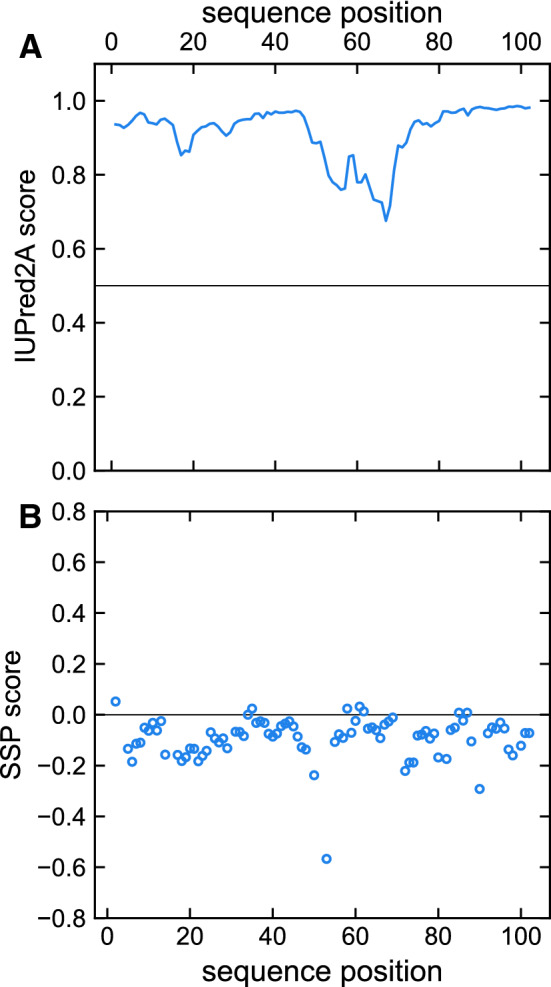
**a** IUPred2A analysis of human DAP1 indicating the intrinsic disorder. The residue-specific IUPred2A score for hDAP1 is indicated as solid line. Disordered segments are indicated by values higher than the default cut-off (0.5), lower values predict structured regions. **b** The sequence specific secondary structure propensity (SSP) scores are depicted (open circles). Values below and above 0 indicate β-sheet and helical-structure propensity, respectively. A SSP score of 1 reflects fully formed helical-structure. Fully formed β-structure is indicated by a SSP value of -1. As recommended for disordered proteins, only ${{^{13}}\text {C}^{\alpha }}$, ${{^{13}}\text {C}^{\beta }}$ and ${{^{1}}\text {H}^{\alpha }}$ chemical shifts were applied and residues immediately preceding prolines were considered when running the SSP script

The analysis of secondary structure content from the assigned chemical shifts by the CSI web server predicts an all coil formation for the entire human DAP1. This supports the observation made from the [${^{1}\text {H}}$, ${^{15}\text {N}}$]-HSQC spectrum. An amino acid sequence based disorder prediction using IUPred2A identifies human DAP1 also as entirely intrinsically disordered (Fig. 4a). In addition, we analyzed the chemical shift data using the secondary structure propensity (SSP) method to reveal potential structural elements (Ja et al. 2006). Even when applying this method no relevant α-helical and β-sheet elements can be detected in the human DAP1 protein (Fig. 4b). The overall content of α-helical and β-sheet elements estimated by the SSP method amounts to 0% and 14%, respectively. From the experimental data and the structural predictions derived, it becomes clear that the human DAP1 is an intrinsically disordered protein under the chosen conditions.

We report the ${{^{13}}\text {C}^{\beta }}$ and ${{^{13}}\text {C}^{\gamma }}$ resonances for all 15 proline residues. All prolines’ ${{^{13}}\text {C}^{\beta }}$ and ${{^{13}}\text {C}^{\gamma }}$ chemical shifts are in the range of 32.2 ppm and 27.5 ppm, respectively. Therefore, it can predicted that all proline residues are in a *trans* conformation (Schubert et al. 2002). The 4 proline residues preceding another proline reveal ${{^{1}}\text {H}^{\alpha }}$, ${{^{13}}\text {C}^{\alpha }}$ chemical shifts at 4.7 ppm and 61.7 ppm, respectively, and can be clearly distinguished from the other prolines, that have ${{^{1}}\text {H}^{\alpha }}$, ${{^{13}}\text {C}^{\alpha }}$ chemical shifts at 4.45 ppm and 63.2 ppm.

We also assigned the chemical shifts of the shorter thrombin cleaved construct (G-2-R93) and would like to emphasize that the ${^{1}\text {H}}$, ${^{13}\text {C}}$ and ${^{15}\text {N}}$ chemical shifts are nearly identical compared to the full-length construct including the purification tag.

## Data Availability

The chemical shift values for the ${^{1}}\text {H}$, ${^{13}}\text {C}$ and ${^{15}}\text {N}$ resonances of the human Death-associated protein 1 have been deposited at the BioMagResBank (https://www.bmrb.wisc.edu) under accession number 50465.
